# Troubleshooting guidewire perforation of the bile duct during endoscopic ultrasound-guided hepaticogastrostomy using the liver impaction technique

**DOI:** 10.1055/a-2704-6702

**Published:** 2025-10-07

**Authors:** Yuichi Takano, Naoki Tamai, Masataka Yamawaki, Jun Noda, Tetsushi Azami, Fumitaka Niiya, Masatsugu Nagahama

**Affiliations:** 1 26858 Division of Gastroenterology, Department of Internal Medicine, Showa Medical University Fujigaoka Hospital, Yokohama, Japan

During endoscopic ultrasound-guided hepaticogastrostomy (EUS-HGS), manipulation of the guidewire is one of the most challenging steps, and guidewire-related bile duct perforation is a potential adverse event 1 2 . When a bile duct perforation does occur, there is no established consensus on management, and repeat puncture is frequently required. The liver impaction technique, first described by Ogura et al., involves withdrawing the puncture needle into the liver parenchyma to prevent guidewire kinking or shearing 3 . This maneuver enables redirection of a guidewire that has entered a peripheral bile duct toward the central duct 4 . We report a case of bile duct perforation that was successfully managed using the liver impaction technique.

The patient was a 90-year-old man with obstructive jaundice due to unresectable pancreatic head cancer. Transpapillary biliary drainage was unsuccessful, and EUS-HGS was performed. Using a convex-array echoendoscope, the B3 intrahepatic bile duct (3 mm) was punctured transgastrically with a 19-gauge needle. The bile duct was small and its lumen was easily collapsed during puncture. Cholangiography confirmed entry into the bile duct, and a 0.025-inch angled guidewire was inserted. However, the wire perforated the bile duct and was misplaced outside the bile duct ( Fig. 1 ).

**Fig. 1:**
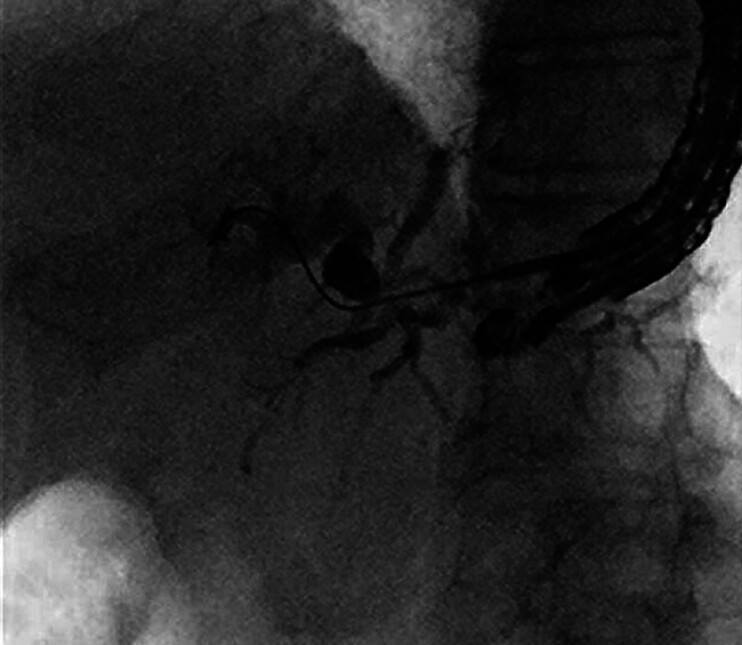
The guidewire perforated the bile duct and was misplaced outside the bile duct during endoscopic ultrasound-guided hepaticogastrostomy.

The puncture needle was then withdrawn into the liver parenchyma. This maneuver restored the collapsed lumen on ultrasound and improved guidewire maneuverability. The guidewire was successfully redirected and advanced into the common bile duct. After catheter insertion, about 30 mL of bile was aspirated. Balloon dilation (3 mm) was performed, followed by placement of a 7-Fr dedicated plastic stent ( Video 1 ). The procedure was completed without adverse events, and the patient’s jaundice improved.

Troubleshooting guidewire perforation of the bile duct during endoscopic ultrasound-guided hepaticogastrostomy using the liver impaction technique.Video 1

In small bile ducts, the lumen often collapses during puncture, increasing the risk of guidewire-related perforation. The liver impaction technique improves guidewire maneuverability and maintains duct patency, making it a useful option for managing guidewire perforation during EUS-HGS.

Endoscopy_UCTN_Code_CPL_1AL_2AD

## Notes

Endoscopy E-Videos https://eref.thieme.de/e-videos
*E-Videos* is an open access online section of the journal *Endoscopy* , reporting on interesting cases and new techniques in gastroenterological endoscopy. All papers include a high-quality video and are published with a Creative Commons CC-BY license. Endoscopy E-Videos qualify for HINARI discounts and waivers and eligibility is automatically checked during the submission process. We grant 100% waivers to articles whose corresponding authors are based in Group A countries and 50% waivers to those who are based in Group B countries as classified by Research4Life (see: https://www.research4life.org/access/eligibility/ ). This section has its own submission website at https://mc.manuscriptcentral.com/e-videos .
